# Spiking time-dependent plasticity leads to efficient coding of predictions

**DOI:** 10.1007/s00422-019-00813-w

**Published:** 2019-12-24

**Authors:** Pau Vilimelis Aceituno, Masud Ehsani, Jürgen Jost

**Affiliations:** 1 Max Planck Institute for Mathematics in the Sciences, Inselstraße 22, 04103 Leipzig, Germany; 2 Max Planck School of Cognition, Stephanstraße 1a, 04103 Leipzig, Germany; 3 Santa Fe Institute, 1399 Hyde Park Road, Santa Fe, NM 87501 USA

**Keywords:** Spiking time-dependent plasticity, Neural code, Predictions, Neural adaptation, Synchronization

## Abstract

Latency reduction in postsynaptic spikes is a well-known effect of spiking time-dependent plasticity. We expand this notion for long postsynaptic spike trains on single neurons, showing that, for a fixed input spike train, STDP reduces the number of postsynaptic spikes and concentrates the remaining ones. Then, we study the consequences of this phenomena in terms of coding, finding that this mechanism improves the neural code by increasing the signal-to-noise ratio and lowering the metabolic costs of frequent stimuli. Finally, we illustrate that the reduction in postsynaptic latencies can lead to the emergence of predictions.

## Introduction

Living organisms need to make accurate predictions in order to survive (Bubic et al. 2010; Hohwy 2013), posing the question of how do brains learn to make those predictions. Early general models based on classical conditioning (Rescorla and Wagner 1972; Miller et al. 1995), as well as mechanistic models explaining the neural substrate for those predictions (Schultz et al. 1997; Heeger 2017) assume that the prediction errors, changes or future rewards feedback to the predicting neural population, similar to supervised or reinforcement learning paradigms that are common in machine learning. However, recent studies have found that sensory neurons without feedback from higher brain areas encode predictive information (Palmer et al. 2015), a finding that has been supported by simulation studies (Sederberg et al. 2018). This implies that a bottom-up process without explicit feedback—similar to unsupervised learning—should also generate predictions.

In this paper, we present such a mechanism by focusing on postsynaptic latency reduction. This is a well-known effect of spiking time-dependent plasticity (STDP) first mentioned by Song et al. (2000) for a single postsynaptic neuron driven by a specific excitatory input pattern. This effect was explored in detail in a simulation study by Guyonneau et al. (2005) who showed that the latency reduction in the target neuron’s firing time is robust to fluctuations in presyanptic input in the form of jitter and Poissonian background noise. They further analyze the STDP effect on a single neuron receiving fixed (among trials) Poissonian spike trains from each presynaptic neuron and showed that by STDP weights of the earliest afferents will be increased, regardless of the firing rate and level of synchrony of the corresponding neurons. Masquelier et al. (2008) showed how a single postsynaptic neuron under the effect of STDP would learn a single frequent excitatory pattern of spikes even in the presence of a strong background noise and how the firing latency in response to this frequent pattern would decrease over learning trials, a finding that was later extended to rate-modulated Poissonian spike trains (Gilson et al. 2011). In another article Masquelier (2018) quantified the performance of a multi-pattern detector neuron in terms of signal-to-noise ratio and showed that STDP results in an optimal SNR in the response of the neuron when some STDP parameters get tuned, see also (Masquelier and Kheradpisheh 2018). Furthermore, Humble et al. (2012) investigated the effect of STDP on a population of neurons with lateral connections and a global winner-take-all mechanism subjected to a longer spatiotemporal input signal. They showed learning leads to the formation of chains of neurons that are responsive to different patterns in the long time input. Similar results have been reported on learning spatiotemporal patterns by variants of STDP rule in Hunzinger et al. (2012) and Sun et al. (2016).

In this article, we revisit this phenomenon at the micro-level with plastic inhibitory neurons added to the previous setups and analyze the effect of latency reduction at the network level, and finally give it meaning as a computational operation as a mechanism for prediction, adding to the works that show that STDP has important computational roles such as formation of memory traces (Klampfl and Maass 2013) or computing Expectation Maximization for probability distributions (Nessler et al. 2013).

The gist of our argument is that latency reduction implies that neurons fire as early as possible for a given input spike train that is repeated very often; as neurons do not differentiate between a specific stimulus and an early clue of such a stimulus—both being part of seemingly the same input spike train—the neurons can, by STDP, fire earlier than the stimulus itself. Furthermore, we expand on the previous studies focused on excitatory neurons to include inhibition and illustrate the parameter regime in which inhibitory plasticity is compatible with latency reduction. However, the latency reduction mechanism has other uses in terms of neural code. First, as neurons fire as early as possible when a stimulus is presented, their spikes will concentrate in a small time window, and thus they are easier to decode. Second, we show that the latency reduction can also lead to a reduction in the number of spikes, which translates as a reduction in metabolic costs for encoding frequent stimuli.

We develop our argument by studying simple models of neurons subject to fixed input spike trains. We use a combination of simulations and mathematical analysis to derive our results, starting from the evolution of a single postsynaptic spike at very short timescales we expand to larger scales that conclude in the emergence of predictions and efficient code at the level of populations of neurons in large timescales.

The rest of this paper is organized as follows. First, we present the models of neurons and STDP in Sect. 2. Second, we study the effects of STDP in a single postsynaptic spike in very small timescales $\sim 10 ms$, focusing on latency reduction and the reduction of the number of postsynaptic spikes in Sect. 3. In Sect. 4, we expand those results to long postsynaptic spike trains , finding that STDP forces postsynaptic neurons to fire only once at the onset of the presynaptic spike train. Afterward, we provide an interpretation of this spike concentration in terms of neural code performance, showing that it leads to lower number of spikes and synchronization. We finalize by illustrating that the same mechanism of latency reduction leads to encoding predictions in Sect. 5.

## Models

### Leaky integrate-and-fire neuron

Neurons are considered to be the basic computational units in the nervous system. Their main feature is the capacity to receive information through electrical impulses, combine this information and send impulses to other neurons. In this paper, we model them as leaky integrate-and-fire neurons with a refractory period (Lapique 1907). In this model, the state of a neuron at a given time is described by its membrane potential *v*(*t*), which evolves according to the equation

1 $$\begin{aligned} \tau _m \dfrac{\mathrm{d} v(t)}{\mathrm{d}t} = -(v(t) - v_0) + i(t), \end{aligned}$$

where $\tau _m = 10\,$ms, $v_0 = -70$ mV. *i*(*t*) is the input to the neuron at time *t*. When the membrane potential reaches a certain threshold $v_{th} = -50\,$mV, the neuron “fires” or “spikes,” meaning that it emits a pulse of current. After firing, the membrane potential is reset to its resting state $v_0$ and kept frozen at this value for a fixed period of time called the refractory period $t_{\mathrm{ref}} = 1\,$ms.

The firing of a neuron generates pulses of current that arrive at other neurons, which in turn update their membrane potentials. If neuron *a* receives the spikes of neuron *b* we will say that there is a synapse going from the second to the first. The receiving neuron is called postsynaptic and the sending neuron is the presynaptic one. This synapse is characterized by a weight $w_{ab}$ and a delay $d_{ab}$ which correspond, respectively, to the gain and the latency that the pulse of neuron *a* goes through before arriving at *b*.

### Input spike trains

Neurons communicate mainly through action potentials or spikes, which are typically modeled as Dirac delta functions, hence the input to a neuron can be described as

2 $$\begin{aligned} i(t) = \sum _n w_n\delta (t-t_n), \end{aligned}$$

where $w_n$ is the weight of the spike, which corresponds to the strength of the synapse from which the spike comes, and $t_n$ is the arrival time of the spike. The weights of the synapses can be positive, if the presynaptic neuron is excitatory, or negative, if it is inhibitory. Through this paper we will assume that every neuron gets an input that will be repeated, meaning that a neuron will always get spikes from different synapses, and although the weights of the synapses might change, the times $t_n$ of the spikes will remain the same in every input repetition. Each synapse comes from a presynaptic excitatory neuron with probability 0.8 or an inhibitory one with probability 0.2.

### Spiking time-dependent plasticity

Networks of neurons learn by modifying the strength of the connections between them. There is a rich literature on what rules those weights follow in biological neurons and their respective implications (Dayan and Abbott 2001). For the purposes of this paper, the neurons will adapt their connections according to the spiking time-dependent plasticity (STDP) paradigm (Sjöström and Gerstner 2010; Gerstner et al. 1996).

In STDP, the weight of a connection is modified depending on the time interval between pairs of pre- and postsynaptic spikes. For every pair, the weight of the synapse is changing according to the equations

3 $$\begin{aligned} \Delta w (\Delta t) = {\left\{ \begin{array}{ll} A_{+}(w) \mathrm{e}^{-\frac{|\Delta t|}{\tau _{s}}} \quad \text { if }\Delta t \ge 0\\ -A_{-}(w) \mathrm{e}^{-\frac{|\Delta t|}{\tau _{s}}} \quad \text { if } \Delta t < 0 \end{array}\right. } \end{aligned}$$

where $\Delta t = t_{\mathrm{post}} - t_{\mathrm{pre}}$ is the time difference between the postsynaptic spike and the presynaptic one, $\tau _{s} = 20\,$ms. Based on previous works (Werner and van Hemmen 2000; Van Rossum et al. 2000), we define $A_+$ and $A_-$ as

4 $$\begin{aligned} \begin{aligned} A_+(w)&= \eta _+ (w_{\max }-w),\\ A_-(w)&= \eta _- (w-w_{\min }) \end{aligned} \end{aligned}$$

where $\eta _- = 0.015,\ \eta _+ = 0.01$, $w_{\max }^e = 10\,$mV and $w_{\min } = 0$. Inhibitory synapses follow the same rules as their excitatory counterparts but with parameters $\eta _- = 0.045$ , $\eta _+ = 0.03$ and $w_{\min }^i = -20\,$mV. This inhibitory kernel has been experimentally observed (Vogels et al. 2013) and its symmetry with respect to its excitatory counterpart will make our analysis simpler, although we shall explore other kernels in “Appendix B”.

### Model limitations and required features

We must note that the models used here are heavy simplifications of real neurons. LIF neurons do not exhibit the rich range of dynamics that real neurons possess(Izhikevich 2004), ion channel kinetics are more complicated than simple Dirac deltas (Chapeau-Blondeau and Chambet 1995) and the STDP model used here cannot account for the evolution of synaptic weights when the frequency of postsynaptic or presynaptic spikes are high (Pfister and Gerstner 2006). However, those models contain the main biologically realistic features that we need for the rest of this study. First, the time constants of the neuron membrane potentials(Gerstner et al. 2014) and the STDP interactions (Bi and Poo 1998) are at least an order of magnitude smaller than the duration of the input spike trains associated to biologically realistic stimuli-evoked spatiotemporal patterns (Rolston et al. 2007; Prut et al. 1998). Second, the neurons have a low firing rate (Roxin et al. 2011). Third, the synapses whose spikes presynaptic spikes arrive shortly before a postsynaptic spike get reinforced, while those arriving afterward get depressed (Sjöström et al. 2008; Pfister and Gerstner 2006). Finally, the homeostatic consideration that firing rates of neurons should not increase widely, which is a natural requirement on metabolic grounds (Turrigiano and Nelson 2004) can easily be incorporated by the depressive term $A_-$. Thus, we will keep these well-known models (Gerstner et al. 2014) on the grounds that they are analytically tractable and qualitatively plausible.

## Evolution of a single postsynaptic spike

In this section, we show that STDP can change individual postsynaptic spikes by reducing their latencies and their number. We will start by presenting simple scenarios with excitatory inputs in which both effects are easy to illustrate, then show how inhibitory synapses can be added to the model, and finally show that those effects can appear in random input spike trains by presenting simulations. It is worth noticing that the time windows in this section are on the order of $\tau _s$ and the number of repetitions of each input pattern will be small.

### Latency reduction

If a fixed train of presynaptic spikes is repeated very often, then the spikes that arrive before the postsynaptic spike get reinforced. This implies that the postsynaptic spike might then be triggered earlier (Song et al. 2000; Gerstner et al. 1996). When this happens, the refractory period of the postsynaptic neuron would prevent a second spike on the original spiking site. However, when the postsynaptic spike happens earlier and earlier, it might lead to a proliferation of spikes by having a new spike appear at the time of the original postsynaptic spike. Following previous literature (Song et al. 2000; Abbott and Nelson 2000; Kempter et al. 2001), to prevent this effect, we assume that long-term depression—the weakening of synaptic weights—is stronger than long-term potentiation—the strengthening of postsynaptic weights.

This is easy to understand in a simple scenario: Considering a very long, excitatory presynaptic spike train which generates a single postsynaptic spike at some time $t_0$. The postsynaptic spike will advance through the spike train, and after some repetitions it will be triggered one presynaptic spike earlier. After this advancement is repeated many times, the postsynaptic spike is triggered at time $t_\infty$, very far (in time) from the place where it was first triggered, so that

5 $$\begin{aligned} t_\infty \ll t_0. \end{aligned}$$

**Fig. 1:**
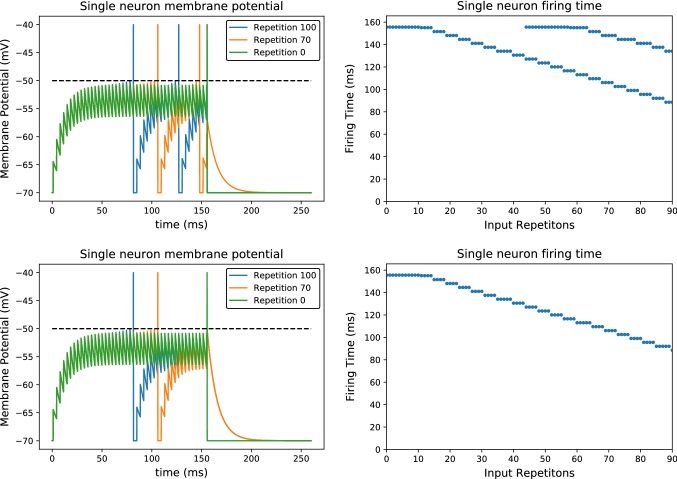
Latency reduction and spike proliferation: We plot the membrane potential (left) and firing times (right) of a postsynaptic neuron that receives a constant train of spikes with inter-spike interval of $3.5\,$ms and strength $5.5\,$mV, from time $t=0\,$ms to $t=150\,$ms, and we add an extra spike at $t=150\,$ms with potential $2\,$mV. The neuron generates a single postsynaptic spike at the original input presentation (Repetition 0). The upper plots reflect the case $\eta _+=\eta _-$, while for the lower ones we picked $\frac{3}{2}\eta _+ = \eta _-$. After an initialization period, the postsynaptic spike moves forward in time at a constant rate. As this happens, a single presynaptic spike will get reinforced proportionally to the $\eta _+$ and dampened proportionally to $\eta _-$. If LTP is equal to LTD, after the postsynaptic spike happens much earlier than before, the membrane potential of the postsynaptic neuron will reach the threshold again. This second postsynaptic spike would move forward in time at the same speed as the strengths of the spikes are left unchanged by the compensation of LTD and LTP (upper plots). In the case where $\eta _+<\eta _-$, the depression compensates the potentiation, so there is no second postsynaptic spike

The membrane potential decays exponentially, meaning that the effect of the postsynaptic spike at time $t_\infty$ on the membrane potential is of order $O(\mathrm{e}^{-\frac{t_0 - t_\infty }{\tau _m}})$, which is negligible. Thus, the membrane potential at time $t_0$ is now only dependent on the presynaptic spikes that are close. If those presynaptic spikes have been left as they where by the passage of the postsynaptic spike, then a new postsynaptic spike will be generated at time $t_0$. To avoid the appearance of this postsynaptic spike it is therefore necessary that the passage of the postsynaptic spike weakens the presynaptic ones. We illustrate this point in Fig. 1 with the functions and parameters that we will use in subsequent sessions.

Note that the argument that we give here is qualitative in nature, in the sense that we simply state that LTD should dominate LTP through the constant $\eta$, but we have not studied how to find that ratio. As this would depend on the exact parameters of the regular spike train—and thus would not be directly generalizable—we will simply assume that the brain operates in a parameter regime in which spikes do not proliferate.

### Late spike disappearance through synaptic noise

If latencies might be reduced, then two postsynaptic spikes that are triggered at distant points in time might become closer in subsequent learning trials. We must then ask what happens to a pair of postsynaptic spikes that occur very close in time. In this section, we show that in the absence of synaptic noise the two spikes can coexist, but random modifications of the presynaptic weights—induced, for instance, by other presynaptic inputs—can lead to the disappearance of the second postsynaptic spike.

There are many possible scenarios that we might consider when we have pairs of postsynaptic spikes in the same neuron: We must consider the time between the two spikes, the movements in time of both of them and the possibility of synaptic noise. The case when two postsynaptic spikes happen originally very close in time is extremely rare—because postsynaptic spikes are sparse. The case where the first postsynaptic spike also moves is not interesting, because the spike will move forward in time, increasing the distance between the two postsynaptic spikes and thus reducing the LTD effect on the second spike—note that the second spike would not move as fast because the presynaptic spikes between them would be depressed by the first. Therefore, we will consider the case where there is an early postsynaptic spike at some fixed time that will remain in place, and a second postsynaptic spike that will initially be triggered very far in time.

The intuition here is that there is a time interval for the second postsynaptic spike, in which the LTD of the first postsynaptic spike would lead to a decrease in the membrane potential of the postsynaptic neuron at the time of the second postsynaptic spike, which could lead to the irreversible disappearance of the second postsynaptic spike or its recession. Outside of this time interval, the second postsynaptic spike will reduce its latency, approaching the early postsynaptic spike and the disappearance zone. In the remaining of this section, we will show that this interval is never reached in a deterministic system but that the addition of noise can enforce this disappearance.

Consider a long presynaptic spike train with presynaptic spikes arriving at $t_0, t_1,\ldots t_N$, which generates two postsynaptic spikes, one at time $t_0$, which is fixed and will appear at every presentation of the spike train, and another one that is originally triggered at $t_N$. For the second spike to disappear, it can either do so at $t_N$ or first advance through the spike train—that means, being triggered at $t_{N-1}$, then at $t_{N-2}$ and so on—and eventually die. For now, we assume that $t_N - t_0 \gg \tau _s$, so that initially the spike at time $t_N$ evolves independently of the spike at time $t_0$, and it would not disappear at $t_N$. Consider now that the input has been repeated long enough so that the second postsynaptic spike is now triggered at $t_i$, and the effects of the STDP generated by the spike at $t_0$ are not negligible to the presynaptic weight $t_{i-1}$, which is associated to the presynaptic spike at $t_{i-1}$. If the postsynaptic spike is originally triggered at $t_i$, then it would move to $t_{i-1}$ only if, after repeating the same input many times,

6 $$\begin{aligned} v(t_{i-1}) = \sum _{k=1}^{i-1} w_k \mathrm{e}^{-\frac{t_k-t_{i-1}}{\tau _m}} \ge v_{th}. \end{aligned}$$

After $v(t_{i-1})$ crosses the $v_{th}$ threshold, the postsynaptic spike at $t_i$ moves to $t_{i-1}$, and thus the time difference between every presynaptic spike at $t\le t_{i-1}$ and the postsynaptic spike is reduced. This naturally implies that the synaptic weights $w_k$ for all $k\le i-1$ increase, thus the postsynaptic spike cannot disappear because the membrane potential at $v(t_{i-1})$ cannot decrease unless the postsynaptic spike moves to $t_{i-2}$. Therefore, with a deterministic input the second postsynaptic spike precedes and stops at an equilibrium distance from the first one. This implies that the weights of the presynaptic neurons arriving at each specific time point in the interval would also reach their equilibrium values.

This argument assumes that presynaptic spike trains are always repeated with fixed spike timings but with weights that are affected by LTP and LTD. This is generally not true, as there are many factors that can introduce stochasticity on the evolution of the weights, such as jitter, the stochastic nature of molecular dynamics on the synaptic cleft and on the neuron membrane.

If we now consider the stability of both postsynaptic spikes with respect to that noise, we easily realize that they are not equal: While the presynaptic spikes that generate the first postsynaptic spike are only subject to LTP and noise, the presynaptic spikes that generate the second spike—which happen necessarily between postsynaptic spikes—are subject to both LTP—from the late postsynaptic spike—and LTD—from the earlier postsynaptic spike—on top of the noise.

This difference implies that the noise can make a postsynaptic spike disappear or recede, either by directly weakening the associated presynaptic weights or strengthening them, so that the postsynaptic spike moves into a region where LTD dominates and it would be later erased or pushed back.

To explain this in the setting that we used before, consider a neuron with a postsynaptic spike at time $t_i$ that would not move to $t_{i-1}$ in the previous deterministic system. However, now the weights evolve by the combined effects of that spike, an earlier postsynaptic spike at time $t_0$ and some noise. The membrane potential at time $t_i$ and after *r* repetitions of the input spike train follows

7 $$\begin{aligned} v(t_{i}) = \sum _{k=1}^{i} w_k \mathrm{e}^{-\frac{t_k-t_{i}}{\tau _m}} + \xi _{t_{i}}, \end{aligned}$$

where $\xi _t$ is the contribution of the random evolution of the weights to *v*(*t*) given by

8 $$\begin{aligned} \xi _{t_i} = \sum _{k=1}^i \delta w_k \mathrm{e}^{-\frac{t_k-t_{i}}{\tau _m}} \end{aligned}$$

where $\delta w_k$ is the deviation of weight $w_k$ from its deterministic evolution; in the case of Gaussian noise, for instance, it would lead to an Ornstein–Uhlenbeck process for the evolution of $v(t_i,r)$ across the number of trial repetitions *r*. Note that the noise is a variable reinitialized at every repetition, but its effects on the weights remain across repetitions.

**Fig. 2:**
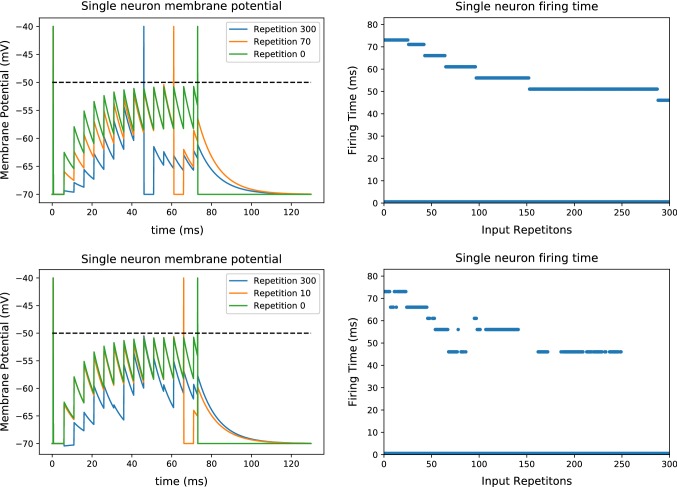
Noise deletes a late spike in a regular presynaptic spike train: We plot the membrane potential (left) and firing times (right) of a postsynaptic neuron that receives a constant train of spikes with inter-spike interval of 5 ms and strength $7.5\,$mV, from time $t=0\,$ms to $t=150\,$ms. We add an extra spike at $t=0\,$ms with potential 5*mV*, which forces a postsynaptic spike at time 0.5 ms. The top row is deterministic, while the bottom row is stochastic as the weights are subject to Gaussian noise with variance of 0.3. Note that, during its existence, the latency of the postsynaptic spike subject to noise decreases faster than its noiseless counterpart

If this postsynaptic spike train is repeated very often, the deterministic part of the weights goes to a fixed value, which is small for $k>i$ and thus $v(t_k)\sim \xi _{t_k}$ for all $k>i$. Thus, under the assumption

9 $$\begin{aligned} \xi _{t_{i}} < v_{th}- \sum _{k=1}^{i} w_k \mathrm{e}^{-\frac{t_k-t_{i}}{\tau _m}} \end{aligned}$$

in a specific trial the second spike will be absent. Subjected to the ever present postsynaptic spike at $t_0$, the weights $w_k$ will decrease for all values of *k* after this trial makes the neuron less likely to fire in the subsequent trials. This negative drift will finally lead to the irreversible disappearance of the postsynaptic spike at $t_i$ or its delay. This is illustrated in Fig. 2.

### Generalization to inhibitory plasticity

Until now we have only considered excitatory neurons. However, in biological systems, inhibitory synapses are also present and show plasticity (Vogels et al. 2013). Naturally, this might compromise the effects described in the previous section, as an inhibitory synapse that gets potentiated could counteract the effects of excitatory STDP. For instance, it might decrease the membrane potential and thus increase the latency of the postsynaptic neuron (Effenberger et al. 2015). Our goal in this section is to find the parameter regime in which the presence of inhibitory plasticity does not compromise the latency decrease and, by extension, the disappearance of postsynaptic spikes.

Intuitively, as long as the STDP in inhibitory synapses is weaker than the STDP in excitatory ones, the latency of postsynaptic spikes would still decrease. The question is then to find a way of measuring “how much weaker” it has to be. To address this issue, we must find a boundary parameter set for inhibitory synapses that guarantees that latency would be reduced, and then we can simply take any parameter set that is between this boundary parameter set and the only excitatory STDP.

To identify the parameter regime in which latency reduction for a single spike appears, we assume that the STDP keeps the balance between excitation and inhibition, in the sense that the average input to a single neuron is maintained constant (Brunel 2000). To maintain this balance, the potentiation of excitatory synapses is compensated by the potentiation of inhibitory synapses. Potentiating all synapses but maintaining the average input leads to the increase in fluctuations of the membrane potential, meaning that the membrane potential preceding a postsynaptic spike would change more around the average, and thus it can still lead to an earlier postsynaptic spike.

Consider a single postsynaptic spike at time $t_{\mathrm{post}}$. For $t<t_{\mathrm{post}}$,

10 $$\begin{aligned} v(t) = \sum _{t_k<t} w_k \mathrm{e}^{-\frac{t-t_k}{\tau _m}}, \end{aligned}$$

and initially $v(t) < v_{th}$. Now we wonder what happens when the weights $w_k$ change, in particular whether the postsynaptic spike will advance, recede or disappear. This depends on the exact values of $w_k$ and $t_k$, so to make more generic statements we are interested in the value

11 $$\begin{aligned} {\mathbb {E}}_r\left[ \Delta t_{\mathrm{post}}\right]= & {} {\mathbb {E}}\left[ t^r_{\mathrm{post}} - t_{\mathrm{post}}\right] \nonumber \\= & {} \left( {\mathbb {E}}\left[ t^r_{\mathrm{post}}\right] -t_{\mathrm{post}}\right) \text {Pr}\left[ \exists s\right] \end{aligned}$$

where *r* accounts for the number of times that the spike train has been repeated, and $\text {Pr}\left[ \exists s\right]$ is the probability that a postsynaptic spike still exists, and the expectations are taken over the presynaptic spike trains—a list of tuples $(w_k,t_k)$ sampled from some predefined distribution—that generate a postsynaptic spike at time $t_{\mathrm{post}}$. In simpler words, we are trying to calculate whether the postsynaptic spike is expected to move forward (${\mathbb {E}}\left[ \Delta t_{\mathrm{post}}\right] (r)< 0$) or backward (${\mathbb {E}}\left[ \Delta t_{\mathrm{post}}\right] (r)> 0$), ignoring the ones that disappeared, if we only have some information about the distribution from which the list of $(w_k,t_k)$ was sampled.

We know that increasing the input excitatory weights can only lead to an earlier postsynaptic spike, because *v*(*t*) can only increase and thus it might reach $v_{th}$ earlier. We will take this a step further and assume that this statement is also true about the average weights, meaning that when the expected input increases, the expected postsynaptic firing time decreases. In more formal terms, we are assuming that ${\mathbb {E}}_r\left[ \Delta t_{\mathrm{post}}\right]$ is a function that decreases monotonically with

12 $$\begin{aligned} {\mathbb {E}}\left[ \Delta _r v(t)\right]= & {} {\mathbb {E}}\left[ \int _{-\infty }^{t}\Delta _r i(t)\mathrm{e}^{-\frac{t-x}{\tau _m}}\mathrm{d}x\right] \nonumber \\= & {} \int _{-\infty }^{t}{\mathbb {E}}\left[ \Delta _r i(t)\right] \mathrm{e}^{-\frac{t-x}{\tau _m}} \mathrm{d}x, \end{aligned}$$

for all $t<t_{\mathrm{post}}$, meaning that if the expected value of $\Delta v(t)$ averaged over all realizations of the input spike train producing a spike at $t_{\mathrm{post}}$ is positive, then ${\mathbb {E}}_r\left[ \Delta t_{\mathrm{post}}\right]$ will be negative.

This assumption, albeit natural, requires some careful consideration. Specifically, we must clarify the distribution over which the expectations are taken, which corresponds to all possible presynaptic spike trains shortly preceding a postsynaptic spike. Those spike trains have fixed timings for every postsynaptic spike under consideration, but are updated systematically because postsynaptic spikes evolve with the input repetitions and the noise. Thus, this distribution considers samples in which a new spike has just appeared or samples where a postsynaptic spike has recently been displaced by a short time.

The subsequent step is to find the conditions that guarantee that ${\mathbb {E}}\left[ \Delta _r v(t)\right]$ increases. A sufficient condition for this to happen is to have

13 $$\begin{aligned} {\mathbb {E}}\left[ \Delta _r i(t)\right] = \Delta _r {\mathbb {E}}\left[ i_e(t)\right] - \Delta _r{\mathbb {E}}\left[ i_i(t)\right] > 0,\ \forall t< t_{\mathrm{post}}\nonumber \\ \end{aligned}$$

where ${\mathbb {E}}\left[ i_e(t)\right]$ is the expected input to the neuron at time *t*, and ${\mathbb {E}}\left[ i_i(t)\right]$, ${\mathbb {E}}\left[ i_i(t)\right]$ is simply its decomposition in inhibitory and excitatory inputs, which gives us

14 $$\begin{aligned} \begin{aligned} {\mathbb {E}}\left[ i_e(t)\right] = \rho _e\int _{0}^{\infty } \mu _{w_e}(w,t) \mathrm{d}w\\ {\mathbb {E}}\left[ i_i(t)\right] = \rho _i\int _{0}^{\infty } \mu _{w_i}(w,t) \mathrm{d}w \end{aligned} \end{aligned}$$

where $\rho _e, \rho _i$ are the rates of incoming spikes and $\mu _{w_e}(w,t), \mu _{w_i}(w,t)$ the probabilities of the weights associated to time *t*.

Thus, to maintain the condition from Eq. 13, we must ensure that the parameters $\mu _{w_e}$, $\mu _{w_i}$, $\eta _+^e$, $\eta _+^i$, $w_{\min }^e$, $w_{\min }^i$ are such that

15 $$\begin{aligned}&\rho _e\int _{0}^{\infty } \Delta w_e(r) \mu _{w_e}(w,t) \mathrm{d}w\nonumber \\&\quad > \rho _i\int _{0}^{\infty } \Delta w_i(r)\mu _{w_i}(w,t) \mathrm{d}w, \end{aligned}$$

where $\Delta w(r)$ are given by the STDP Eq. 3 over many repetitions—counted by *r*—of the input spike train. We will now find a parameter regime in which this holds by finding its boundary. In other words, we are interested in the parameter set in which the excitatory increase in weight exactly matches the inhibitory increase in weight, which for the time constants of inhibition being equal to that of excitation leads us to the condition

16 $$\begin{aligned}&\rho _e\int _{0}^{\infty } \Delta w_e(r) \mu _{w_e}(w,t) \mathrm{d}w\nonumber \\&\quad = \rho _i\int _{0}^{\infty } \Delta w_i(r)\mu _{w_i}(w,t) \mathrm{d}w. \end{aligned}$$

Note that it is not enough to find two weight distributions $\mu _{w_e}$, $\mu _{w_i}$ where

17 $$\begin{aligned}&\rho _e\int _{0}^{\infty } A_+^e(w_e) \mu _{w_e}(w,t) \mathrm{d}w \nonumber \\&\quad = \rho _i\int _{0}^{\infty } A_+^i(w_i)\mu _{w_i}(w,t) \mathrm{d}w, \end{aligned}$$

because this would only work for the first input repetition. We have to ensure that even after STDP changes the distribution, the equality holds. Since there are typically fewer inhibitory synapses than excitatory ones, we correct the input rates and STDP parameters by the ratio

18 $$\begin{aligned} \alpha = \dfrac{\rho _i}{\rho _e} \end{aligned}$$

that is also intrinsic to the probability distributions

19 $$\begin{aligned} \alpha \mu _{w_i}\left( \alpha x,t\right) = \mu _{w_e}(x,t) \ \forall x,t, \end{aligned}$$

and the STDP parameters

20 $$\begin{aligned} \alpha A_+^i(\alpha x) = A_+^e(x)\ \forall x. \end{aligned}$$

If these properties are satisfied,by a simple change of variable, we can show that

21 $$\begin{aligned} \begin{aligned}&\rho _e\int _{0}^{\infty } A_+^e(x) \mu _{w_e}(x,t) \mathrm{d}x \\&\quad = \dfrac{1}{\alpha } \rho _i \int _{0}^{\infty } \alpha A_+^i(\alpha x) \alpha \mu _{w_i}\left( \alpha x,t\right) \frac{1}{\alpha } \mathrm{d}(\alpha x)\\&\quad = \rho _i\int _{0}^{\infty } A_+^i(y)\mu _{w_i}(y,t) \mathrm{d}y. \end{aligned} \end{aligned}$$

Furthermore, if we take a pair of inhibitory and excitatory weights such that $w_e = \alpha w_i$ we have that after applying the STDP rule,

22 $$\begin{aligned} \alpha w_i \rightarrow \alpha (w_i + A_+^i(w_i))= & {} \alpha w_i + \alpha A_+^i(\alpha w_e) \nonumber \\= & {} w_e + A_+^e( w_e) \leftarrow w_e, \end{aligned}$$

meaning that the weight probability changes in such a way that

23 $$\begin{aligned} \mu _{w_e}'\left( x + A_+^e(x),t\right)= & {} \mu _{w_e}\left( x,t\right) = \alpha \mu _{w_i}\left( \alpha x,t\right) \nonumber \\= & {} \alpha \mu _{w_i}'\left( \alpha \left( x + A_+^i(x)\right) ,t\right) , \end{aligned}$$

where $\mu _{w_e}'$ and $\mu _{w_i}'$ are the weight distributions after STDP has acted once. Thus, if Eq. 19 holds at some point, it will also hold for all subsequent iterations of the input spike pattern.

Thus, we have found a set of conditions that satisfy Eq. 19 at $r=0$ and for any subsequent $r>0$ for the case where the postsynaptic spike does not change during the *r* repetitions. Notice that the self-consistency of this condition does not make any assumptions about the learning constant or the $\Delta t$ dependent term on STDP, or even its sign, it only requires that the expected increase—or decrease—in excitatory input is matched by the expected increase—or decrease—in inhibitory input. In particular, this symmetry does not change if the postsynaptic spike advances, because the STDP kernel has the same ratio of potentiating inhibitory and excitatory synapses. In other words, when a postsynaptic spike changes places before the *r*th input repetitions, the variance of the input before the postsynaptic spike still increases and, conversely, the variance after the postsynaptic spike decreases.

Now, we have a large set of parameters in which latency reduction is expected to happen. Any STDP parameters for which $\alpha A_+^i(\alpha x) < A_+^e( x)$ combined with Eq. 19, or distribution of weights with $\alpha \mu _{w_i}\left( \alpha x,t\right) < \mu _{w_e}(x,t)$ with Eq. 20, or both cases combined.

It is worth noticing that in the case when all the equalities Eqs. 19 and 20 are met, we would still expect the latency to decrease. The reason is that even if

24 $$\begin{aligned} {\mathbb {E}}\left[ \Delta _r v(t)\right] = {\mathbb {E}}\left[ \Delta _r i(t)\right] = 0, \end{aligned}$$

the variance of *v*(*t*) increases. More explicitly,

25 $$\begin{aligned} \begin{aligned} \Delta _r\text {Var}\left[ v(t)\right]&= \Delta _r \int _{-\infty }^{t}\text {Var}\left[ i(t)\right] \mathrm{d}t\\&= \Delta _r \int _{-\infty }^{t}\left( {\mathbb {E}}\left[ i^2(t)\right] - {\mathbb {E}}\left[ i(t)\right] ^2\right) \mathrm{d}t \\&= \Delta _r \int _{-\infty }^{t}{\mathbb {E}}\left[ i^2(t)\right] \mathrm{d}t = \int _{-\infty }^{t} \Delta _r {\mathbb {E}}\left[ i_\mathrm{e}^2(t)\right] \mathrm{d}t \\&\quad + \int _{-\infty }^{t} \Delta _r {\mathbb {E}}\left[ i_i^2(t)\right] \mathrm{d}t \end{aligned}\nonumber \\ \end{aligned}$$

where the term ${\mathbb {E}}\left[ i(t)\right] ^2 = 0$ by the symmetry of the weights and it is maintained at zero by the symmetry of the STDP. Since we are only concerned with $t<t_{\mathrm{post}}$, STDP potentiates both inhibitory and excitatory synapses, so

26 $$\begin{aligned} \begin{aligned} \Delta _r {\mathbb {E}}\left[ i_i^2(t)\right] ,\Delta _r {\mathbb {E}}\left[ i_\mathrm{e}^2(t)\right] > 0 \end{aligned} \end{aligned}$$

and therefore the variance increases. Naturally, if the variance of a certain distribution increases while keeping its mean constant, then the probability of reaching a value higher than some threshold—$v_{\mathrm{th}}$—also increases.

The approach outlined here can be also used for other STDP kernels. While the symmetry in the excitatory and inhibitory STDP kernels might not exist for some choices of inhibitory and excitatory plasticity, the approach can, in principle, still be used by making sure that the mean or the variance of the inputs to a neuron would grow before each postsynaptic spike

The nonproliferation of spikes can be derived by a similar argument, although in this case the mean or the variance (or both) of the presynaptic input to the postsynaptic neuron will decrease due to the depressive nature of STDP for $t>t_{\mathrm{post}}$. In general, the idea of having the depression stronger than the potentiation would still work, as long as the depression of inhibitory synapses is weaker or equal than that of excitatory synapses. As this calculation is essentially the same as the one we just presented, we will skip it.

### Numerical verification for random input spike trains

The examples presented to illustrate the latency reduction and the disappearance—or delay—of late postsynaptic spikes were simple, so we must now extend them to a more general case. To do so, we simulated spike trains where the times of the presynaptic spikes are randomly sampled at the beginning and then fixed for every subsequent repetition, including only excitatory or excitatory and inhibitory STDP, noise and the presence of an earlier postsynaptic spike. The results are presented in Table 1 and agree with our previous conclusions: A single postsynaptic spike tends to reduce its latency, if there are multiple postsynaptic spikes in a short time window the later ones tends to disappear, and the presence of noise increases those effects. Note that we have not included jitter or probabilistic presynaptic spikes, choosing instead to have noise directly on the weight evolutions. As both cases have been addressed before (Guyonneau et al. 2005) with similar conclusions, we shall not repeat them here.

**Table 1:** Effects of STDP on short random spike trains: We explored the effects of STDP on the postsynaptic spike train of a neuron receiving 8 excitatory and 2 inhibitory presynaptic spikes arriving at uniformly sampled times on the interval $\left[ 0, 40\,\mathrm{ms}\right]$ and the stimulus is repeated 100 times

STDP type	Noise var.	Spike at $t=0$	Spike count increase (%)	Spike count decrease (%)	Spike latency increase (%)	Spike latency decrease (%)	Average latency change (ms)
E and I	0	No	0.1	13.5	5.2	51.9	$-$2.7
E and I	0.2	No	1	30.6	13.8	23.1	$-$1.5
E	0	No	1.4	0.0	0.5	81.4	$-$3.1
E	0.2	No	4.2	11.3	16.2	32.6	$-$0.34
E and I	0	Yes	0.0	8.3	28.8	22.1	3.2
E and I	0.2	Yes	0.6	21.5	33.1	13.1	2.8
E	0	Yes	5.2	10.8	15.9	31.3	0.1
E	0.2	Yes	2.8	12.9	27.2	18.7	3.3

The first three columns determine the setup: The STDP Type indicates if STDP was active for excitatory presynaptic neurons only (E) or for inhibitory as well as excitatory (E and I) with the inhibitory STDP having the parameters to exactly compensate the excitatory one as presented in Sect. 3.3, the second column indicates the variance of the Gaussian noise added to every weight at every stimulus repetition, and the third column indicates whether we added a postsynaptic spike at the beginning of the time window. The remaining columns explain the results: The fourth one indicates the percentage of spike trains in which new postsynaptic spikes appeared, the fifth one the percentage of spike trains in which a spike disappeared, the sixth one the percentage of spike trains in which a single postsynaptic spike (not counting the imposed one at $t=0$) happened later after learning, the seventh one corresponds to the postsynaptic spike happening earlier, and the last one is the average latency change of the postsynaptic spikes (here we only accounted for the cases where there was a single postsynaptic spike at the beginning and at the end of the learning). We calculated the percentages and averages from 1000 randomly generated spike trains in which a single postsynaptic spike was triggered at the beginning of the training. The results clearly show that in all cases spike latencies tend to decrease when no spike is placed at $t=0$, and increase otherwise. Naturally, adding noise or inhibitory plasticity increases the percentage of spikes that disappear. Similarly, adding the initial spike increases the number of disappeared spikes

So far we have only considered effects on small time scales, meaning that there were only a few spikes on a time interval of the order of $10\,$ms, and the postsynaptic spike train would evolve over a few repetitions, on the order of $20\,$ms. This leads us to the conclusion that, with plausible assumptions on the parameters of our model, an individual postsynaptic neuron will fire a specific postsynaptic spike earlier after many repetitions of the same presynaptic spike train and that if two postsynaptic spikes are close in time, then the later one could disappear.

## Postsynaptic spike train

Now, we study the effects of the previously described phenomena, which act on small temporal scales and affect only one or two postsynaptic spikes, for a population of postsynaptic neurons, each one receiving many presynaptic spike trains happening over time scales much larger than $\tau _m$ or $\tau _s$. Specifically, we will explore the latency reduction and suppression or delaying of late postsynaptic spikes and the change in the postsynaptic spike distribution.

Before studying those effects, we must validate some of the assumptions that we made in the previous section. In particular, we assume that all the input spikes came from different synapses, which allowed us to treat the weights of all presynaptic spikes as independent. This is a valid assumption when we are considering short time intervals, as the sparsity of presynaptic firing and the existence of refractory periods implies that a single synapse would typically not fire more than once during a short presynaptic spike train. However, when there is a long presynaptic spike train, a presynaptic neuron might contribute to that spike train more than once, thus our assumption might be invalid and the phenomena described in the previous section might not appear. To ensure that the phenomena of latency reduction and late spike disappearance are still present in long spike trains, we use a combinatorial argument and count the number of synapses that might evolve in a non-trivial fashion, which we present in “Appendix A”.

We can now consider the first time that an input presynaptic spike train is presented. Every neuron starts at $v(0) = 0$ and then its membrane potential will change depending on its inputs. As the input spike train consists of independent spikes with independent weights, the times of the first spike have a probability distribution $f_0^{1}(t)$ with support on $t>0$, which depends on the parameters of the input spike train. After spiking, every neuron resets its membrane potential to zero, and thus the distribution of inter-spike intervals $f_0^{\mathrm{ISI}}(t)$ follows

27 $$\begin{aligned} f_0^{\mathrm{ISI}}(t) = f_0^{1}(t-t_{\mathrm{ref}}). \end{aligned}$$

**Fig. 3:**
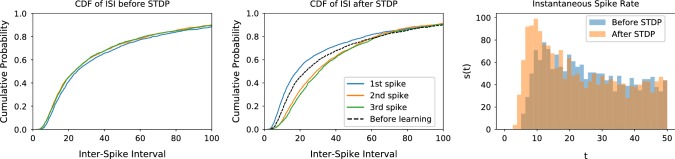
Evolution of the spike train: We plot cumulative probability distribution of the time of the first spike, the inter-spike interval when a presynaptic spike train is presented for the first time (left) and after many repetitions (center) and the number of spikes per bins of $4\,$ms on the first $50\,$ms of a spike train (left). We simulate 2000 neurons each receiving a presynaptic spike train lasting $600\,$ms with 200 presynaptic spikes, both inhibitory and excitatory, and whose arrival time is uniformly sampled. Every synapse evolves through STDP and being subject to both the fixed spike train with probability 0.33 and a random pair of pre- and postsynaptic spikes with $t_{\mathrm{post}} - t_{\mathrm{pre}} \in \left[ -20\,\text { ms}, 20\,\text {ms}\right]$ with probability 0.66. We plot the time of the first spike (blue) and the inter-spike interval for second, third and fourth spikes, but subtracting the refractory period to have a pertinent comparison with the first spiking time. We can see that initially the first spike time is the same as the inter-spike interval for all the spikes, but after STDP is applied the average time of the first spike reduces, implying that the blue line moves to the left with respect to the time before learning (in the black dotted line) while the average inter-spike intervals increase, thus moving the curves to the right. This changes the distribution of spikes to have more of them concentrated in the beginning of the spike train (color figure online)

After the input has been repeated many times, the distribution of postsynaptic spikes changes to $f_\infty ^1$ and $f_\infty ^{\mathrm{ISI}}$, respectively. Specifically, the first spikes reduce their latency on average and thus move closer to $t=0$, while the inter-spike intervals increase, due to the depressive effect of postsynaptic spikes that repels or eliminates late postsynaptic spikes. Therefore,

28 $$\begin{aligned} \begin{aligned} F_\infty ^{1}(t)&= \int _{0}^{t} f_\infty ^{1}(x) \mathrm{d}x \ge \int _{0}^{t} f_0^{1}(x)\mathrm{d}x = F_0^{1}(t)\\ F_\infty ^{\mathrm{ISI}}(t)&= \int _{0}^{t} f_\infty ^{\mathrm{ISI}}(x) \mathrm{d}x \le \int _{0}^{t} f_0^{\mathrm{ISI}}(x)\mathrm{d}x = F_0^{\mathrm{ISI}}(t) \end{aligned} \end{aligned}$$

where $F_\infty ^{1}, F_\infty ^{\mathrm{ISI}}, F_0^{\mathrm{ISI}}$ and $F_0^{F}$ are the cumulative probability distributions of the inter-spike intervals and first spikes, respectively. This is illustrated in Fig. 3 showing that indeed the first spikes move forward through STDP and the later spikes are more separated, which is consistent with the results from previous sections.

It is worth noting that our results are only valid for the specific case where the plasticity rule potentiates the presynaptic spike to a neuron before its postsynaptic spikes and depresses those afterward. As there is a zoo of possible time-dependent rules, we performed a short overview of the effects of those rules in “Appendix B,” finding that in a wide range of variants our results still hold. Another important feature that we have to consider is the addition of recurrent connections, which we address in “Appendix C”.

For the next section, it will be convenient to look at the instantaneous firing rate, which is obtained by accumulating the times of all spikes.

29 $$\begin{aligned} s(t) = \lim \limits _{\Delta t\rightarrow 0} \sum _{k=1}^{\infty } \dfrac{\text {Pr}\left[ t_k\in \left[ t,t+\Delta t \right] \right] }{\Delta t} \end{aligned}$$

where $t_k$ is the time of the *k*th spike. Since the time of the *k*th spike is the sum of the inter-spike intervals of the first $k-1$ spikes and the first spike, and the probability of a sum is given by the convolution of the probability distributions, we can rewrite the previous function as

30 $$\begin{aligned} s(t)= & {} \left( f^1 + f^1*f^{\mathrm{ISI}} + f^1*f^{\mathrm{ISI}} *f^{\mathrm{ISI}} + \cdots \right) (t) \nonumber \\= & {} \left( f^1*\sum _{k=0}^{\infty }\left( f^{\mathrm{ISI}}\right) ^{*k} \right) \end{aligned}$$

where $*$ is the convolution operator, $^{*k}$ is the convolution power. Note that $f^1$ and $f^{\mathrm{ISI}}$ depend on how many times the input has been repeated. We will refer to the subindex 0 and $\infty$ to refer, respectively, to the cases where the presynaptic spike train is presented for the first time or when it has been presented many times.

The postsynaptic spike trains generated by neural populations are instantiate codes that transmit information about presynaptic spikes to other neurons. As STDP is a learning mechanism that modifies the postsynaptic spike train, we expect that it should improve this encoding. Each input stimulus triggers spikes in a certain neural population, and every neuron in that population has a certain performance associated to it, the two most common performance measures being energy consumption and resistance to noise (Rappaport 1996).

If we take the number of postsynaptic spikes generated by the neural population as a proxy for the metabolic costs of encoding a stimulus, then we would expect that number to decrease as the stimulus is presented more often, so that the encoding of common stimuli incurs less metabolic costs.

**Fig. 4:**
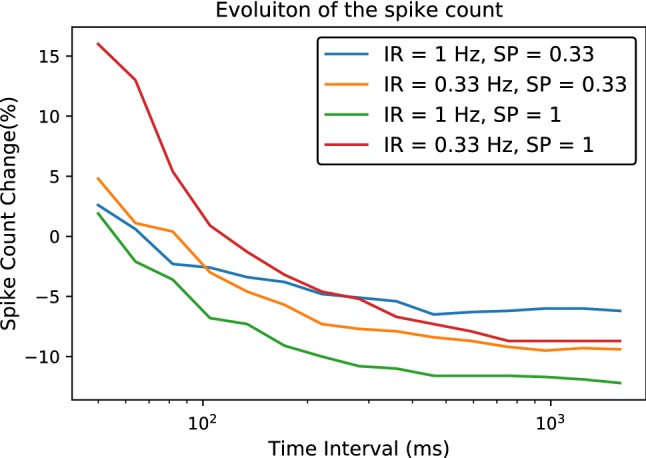
Spike count evolution: We simulated spike trains of various lengths for different parameters of the input rate (IR) and the probability that the stimulus is presented (SP), and when it is not we induce a random pair of pre-postsynaptic spikes in every synapse with a $t_{\mathrm{post}} - t_{\mathrm{pre}} \in \left[ -20\,\text {ms}, 20\,\text {ms}\right]$. In either case we investigate the change in the number of spikes. As we can see, for long spike trains the inter-spike-intervals increase and thus the number of spikes decreases. For short spike trains, on the other hand, there is at most one spike that can fit, so the inter-spike intervals are irrelevant. Furthermore, the spikes in such short intervals are self-maintained when STDP is present: If a spike appears and disappears when the presynaptic weights evolve randomly, the presence of a postsynaptic spike will potentiate those weights, hence the spike will be maintained, implying that STDP increases the number of spikes in short time intervals

To evaluate how the number of spikes evolves, we consider the evolution of the first spike and inter-spike-interval cumulative probability distributions from Fig. 3. On one hand, the fact that the first spike moves forward implies that there will be more spikes concentrated on a small region at the beginning, so if we consider a very short time interval the concentration of spikes will increase. However, as we increase the length of the stimulus, the average distance between spikes will start to depend mostly on the inter-spike interval, implying that the spike density will be lower. In more formal terms, the number of spikes is given by the integral

31 $$\begin{aligned} S= & {} \int _{0}^{T} s(t) \mathrm{d}t = \int _{0}^{T} f^1(t)\mathrm{d}t \nonumber \\&+ \int _{0}^{T} f^1 *\sum _{k=1}^{\infty }\left( f^{\mathrm{ISI}}\right) ^{*k} \mathrm{d}t , \end{aligned}$$

which is dominated by the first term when *T* is small and by the second term when *T* is large. This can be quantified by the ratio in the decrease in spikes

32 $$\begin{aligned} \dfrac{S_\infty - S_0}{S_0}, \end{aligned}$$

where $S_0$ is the number of spikes before STDP and $S_\infty$ is the number afterward. Naturally, there are many parameters that affect the change in the number of spikes, in particular the length of the stimuli and the input rate or how often the input is presented with respect to other stimuli, which are shown in Fig. 4. In general, in short time intervals at most one spike would be present, thus the disappearance of second spikes induced by the depressive side of STDP does not play a role; at the same time, the spikes that would appear by the fluctuations in input weight, and which would simply disappear by the same process if STDP was not present, remain. Hence, in that case the number of spikes increases, while for long spike intervals the number of spikes decreases.

It is worth noticing that the reduction in the number of spikes that we observe in Fig. 4 does not correspond to the reduction in spike count that STDP induces in Poissonian spike trains. We tested this by checking how a Poissonian spike train with the same STDP parameters and the same weight distribution and input rate as in Fig. 3 changed, and we found that this leads to an increase of $10 \%$ in the number of postsynaptic spikes because excitatory presynaptic spikes tend to induce postsynaptic spikes, thus the excitatory weights systematically increase.

**Table 2:** Synchrony evolution: We simulated spike trains with different values of the presynaptic input rate (IR) and the probability that the stimulus is presented compared to random pair of spikes per synapse (SP), and then measured the change in $\phi$ taking a time window of 100*ms* and using 1000 neurons. As we can see, the synchronization always increases

	SP = 0.33		SP = 1	
	IR = 0.33	IR = 1	IR = 0.33	IR = 1
L = 2	$2.1 \rightarrow 2.7$	$2.8 \rightarrow 3.9$	$2.3 \rightarrow 3.4$	$2.9 \rightarrow 4.9$
L = 5	$1.8 \rightarrow 2.4$	$2.4 \rightarrow 3.0$	$2.0 \rightarrow 3.0$	$2.5 \rightarrow 3.6$
L = 10	$1.7 \rightarrow 2.0$	$1.9 \rightarrow 2.1$	$1.9 \rightarrow 2.6$	$1.9 \rightarrow 2.3$

Besides the number of spikes, it is also interesting to note how the distribution of those spikes change. Specifically, as the first spikes move forward, the spike train will become more synchronous as the distribution of spiking times becomes sharper, as we can see in Fig. 3, where the postsynaptic spike train has a peak of spikes that grows after STDP is applied. We quantify this by counting the highest concentration of spikes in a small time window of size *L* with respect to the total number of spikes, which can be written as

33 $$\begin{aligned} \phi = \dfrac{\max _t\int _{t}^{t+L}s(t)\mathrm{d}t}{S}\dfrac{T}{L}, \end{aligned}$$

where *T* is the time interval for the full stimulus such that $\frac{S}{T}$ is the average spike rate and $\frac{\max _t\int \nolimits _{t}^{t+L}s(t)\mathrm{d}t}{L}$ is the highest rate in a time window of length *L*. For a random spike train, the highest rate of spikes in a time window of length *L* would be similar to the average firing rate, corresponding to a $\phi \approx 1$. However, if many spikes concentrate in a small time window, the spike trains are synchronized and we obtain a high value of $\phi$. The results of simulations for various parameters are presented in Table 2, where the increase in $\phi$ can be easily seen.

**Fig. 5:**
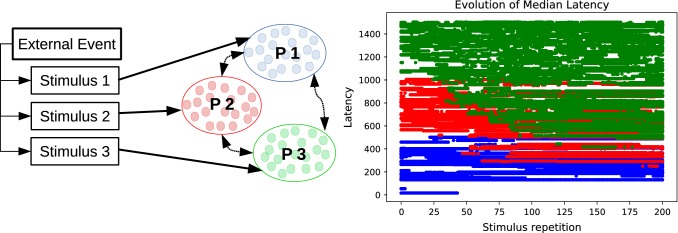
Encoding predictions: schema for the emergence of predictions (left) and firing latencies of neurons in encoding population (right): An external event creates three stimulus that trigger all the neurons in corresponding distinct neural populations $P1, \ P2,\ P3$ with the stimuli inducing spikes during the intervals $[0\,\text {ms},500\,\text {ms}]$ for *P*1, $[500\,\text {ms},1000\,\text {ms}]$ for *P*2 and $[1000\,\text {ms},1500\,\text {ms}]$ for *P*3, respectively. The three populations, with $N=50$ neurons each, also have synapses between them with delays sampled from a uniform distribution between $d_{PiPj} \in [1\,\text {ms},5\,\text {ms}]$. Originally, almost all neurons in each population fire only after receiving inputs from their respective stimuli, but after the external event is repeated very often, the inter-population connections become strong enough to trigger some spikes before the stimulus is received (color figure online)

## The emergence of predictions

When a group of neurons encodes a stimulus we mean that those neurons fire when the stimulus is presented. However, the neurons themselves are not aware agents and do not know anything about that stimulus; they simply receive a spike train that is strong enough to trigger their spiking. From the point of view of an encoding neurons, there is no difference between the stimulus-induced presynaptic spike train and any other input spike train that always precedes the stimulus.

Combining this observation with the results from previous sections showing that neurons will fire at the onset of a frequent input spike train, we can conclude that a neuron that “encodes” a stimulus can start firing before the stimulus is presented if another stimulus appears before it. As an illustrative example, imagine listening to a melody. Different parts of the melody trigger the activity of different groups of neurons in the same area of the brain. If the melody is repeated very often, the neurons *P*1 that react to an early part of the melody will systematically fire before the neurons *P*2 that react to a later part. As the melody is repeated, neurons in *P*2 will always fire after receiving spikes from neurons in *P*1 and thus the synapses from *P*1 to *P*2 will be reinforced. Eventually, the reinforced synapses might trigger spikes in *P*2 before the late part of the melody sounds. This can be extended to more populations encoding more stimuli, and thus the whole melody is encoded through simultaneous activity of all the neurons which originally encode only separate notes. This is illustrated and simulated in Fig. 5.

It is important to notice here that the predictions that we mention here are restricted to stimuli sequences that can be identified from the first input, meaning that we are not addressing the case of two sequences of stimuli which start activating the same neural population and then go on to activate different populations. If we have two possible stimuli sequences which start equally, STDP would force some neurons associated to both possible sequences fire at the onset of the stimuli, meaning that the system would learn that both sequences might follow. However, the differentiation of the two sequences can only be done when the two diverge, so the system must learn to maintain memory traces of the stimuli, a process that can also be implemented by STDP with lateral inhibition (Klampfl and Maass 2013).

## Discussion

In this paper, we start by analyzing and expanding previous findings on latency reduction (Song et al. 2000; Guyonneau et al. 2005). Then, we extend them to trains of spikes and show that those mechanisms lead to encoding the more common inputs with less spikes while concentrating the remaining spikes in smaller time windows. This leads us to the conclusion that STDP reduces the amount of spikes used to encode frequent stimuli, in line with the idea that metabolic efficiency is one of the guiding principles of the brain (Hasenstaub et al. 2010; Laughlin 2001). The same phenomena also synchronize spikes by concentrating them in small time windows. Following the idea that synchronization improves communication between neuronal assemblies (Singer 2011; Fries 2005; Von Der Malsburg 1994), the fact that synchronization is induced by STDP indicates that its effects can be interpreted in communication terms. Finally, we show that the latency reduction can explain how the nervous system learns to forecast even without any feedback.

This study is another example of how simple, well-known plasticity rules that are present at synaptic level lead to modifications that are advantageous at the organism level. Furthermore, the fact that the same mechanism improves the neural code and creates predictions might explain how the ability of the brain to make predictions—which is one of the core problems in cognitive science—could have emerged as a consequence of evolutionary pressures on metabolic cost and information transmission.

Naturally, our work is also interesting for researchers in machine learning, as it shows that Hebbian learning rules, which are classically used to infer or reinforce correlations (Dayan and Abbott 2001), can be used to find predictions by adding a temporal asymmetry in the synaptic plasticity kernel. Furthermore, the fact that the same mechanism gives rise to predictions and coding efficiency is another example of the intimate relationship between machine learning and coding (MacKay et al. 2003), thus it might be interesting for information theorists.

The results exposed here also open new questions. The effects of latency reduction in networks of neurons—in particular recurrent ones—or the potential use of this prediction capabilities of STDP for machine learning require further study but could be useful extensions. However, the most immediate question is whether this unsupervised process is used in the nervous system. An experimental study should identify the neurons that encode two temporally correlated stimuli and follow the evolution of latencies as the stimuli are repeated, while simultaneously ensuring that this process was due to STDP alone without interference of reward systems that have been previously proposed.

## Figures and Tables

**Fig. 6:**
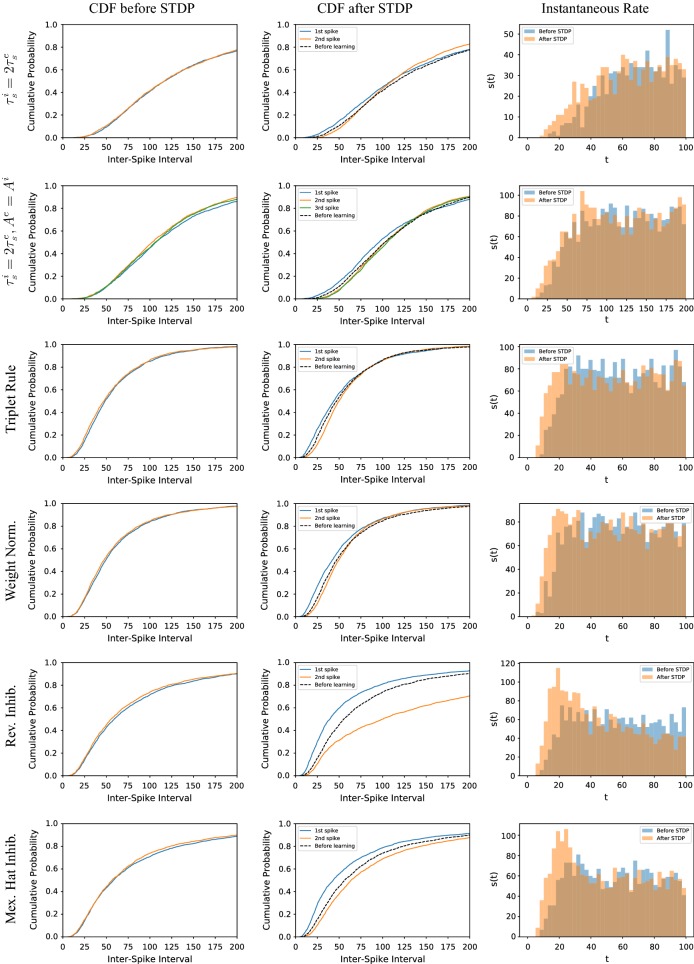
Effects of alternative learning rules: We tested different timing-based learning rules with the same setup as in Fig. 3: 2000 neurons, 200 presynaptic spikes in $600\,$ms, and the input is presented with probability 0.33, with a random pre-postsynaptic pair being presented otherwise. We plot the cumulative probability distribution of the time of the first spike, the inter-spike interval when a presynaptic spike train is presented for the first time (left) and after many repetitions (center) and the number of spikes per bins of $4\,$ms on the first $100\,$ms of a spike train (left)

**Fig. 7:**
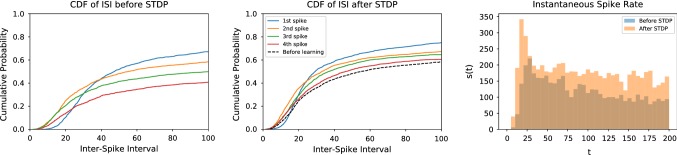
Evolution of the spike train with strong recurrent connections: We plot cumulative probability distribution of the time of the first spike, the inter-spike interval when a presynaptic spike train is presented for the first time (left) and after many repetitions (center) and the number of spikes per bins of $4\,$ms on the first $200\,$ms of a spike train (left). We simulate 2000 neurons each receiving a presynaptic spike train lasting $400\,$ms with 150 presynaptic spikes, both inhibitory and excitatory, and whose arrival time is uniformly sampled, and 15 recurrent ones. Every synapse evolves through STDP and being subject to both fixed spike train with probability 0.33 or a random pair of pre- and postsynaptic spikes with probability 0.66. We plot the time of the first spike (blue) and the inter-spike interval for second, third and fourth spikes, but subtracting the refractory period to have a pertinent comparison with the first spiking time (color figure online)

## Data Availability

The code used in this work is available for download through github under the following link:https://github.com/pvili/SpikingTimeDependentPlasticity

## Postsynaptic spikes evolve independently

The problem with having multiple spikes per presynaptic neuron is that all of the presynaptic spikes coming from the same synapse have the same weight, and therefore when a postsynaptic spike is close to one of those presynaptic spikes, all of the presynaptic spikes that come from that synapse will undergo the same weight modifications. There are two scenarios when this would be a problem:

1. A single synapse undergoes STDP from two or more different spikes: If there are two postsynaptic spikes, affected by their respective presynaptic spikes, but some of those presynaptic spikes come from the same synapse, the resulting weight change from STDP would be a combination of the effects of both postsynaptic spikes. This is undesirable as the effects could be opposite: one postsynaptic spike could induce depression while the other potentiation, and thus the evolution of one of the presynaptic spikes would not evolve as our STDP rule predicts.
2. A new postsynaptic spike appears spontaneously from STDP: Typically, STDP applies only when there exists a postsynaptic spike. However, if some synapses are very strong due to STDP, and those synapses have spikes that are close together, they could generate a new postsynaptic spike. This would automatically generate pairs of presynaptic spikes that are affected by two postsynaptic spikes simultaneously (thus we would be in the previous case). Furthermore, the spontaneous generation of new postsynaptic spikes is itself problematic.

Consider *M* presynaptic neurons which fire with a rate $\lambda$, and a postsynaptic neuron that receives them with a rate $\rho = M\lambda$ during a time interval of length *T*, generating $s_{\mathrm{post}}$ postsynaptic spikes. Furthermore, each one of those postsynaptic spikes imposes STDP that affects the presynaptic spikes that are close to it. For simplicity, we will assume that the noticeable effect on the presynaptic spikes is restricted to a time window of size $l\tau _S$ where *l* is a small integer number.

We start by studying case (1). If we have $s_{\mathrm{post}}$ postsynaptic spikes, then the effects of STDP are noticeable for

34 $$\begin{aligned} t_a = l\tau _S s_{\mathrm{post}} \end{aligned}$$

milliseconds in which all presynaptic spikes should come from different synapses. Given that the arrival times of each spike are uniform on the whole interval, the expected number of presynaptic neurons that fire in that interval more than once is given by

35 $$\begin{aligned} N\sum _{k=2}^{\infty } \dfrac{(\lambda t_a)^k\mathrm{e}^{-\lambda t_a}}{k!}= N\left( 1 - \mathrm{e}^{-\lambda t_a} - \lambda t_a \mathrm{e}^{-\lambda t_a}\right) , \end{aligned}$$

and by a Taylor expansion to order two,

36 $$\begin{aligned}&{\mathbb {E}}\left[ \#1\right] \approx N \left( 1- \lambda t_a + \dfrac{\lambda ^2 t_a^2}{2}+ \lambda t_a - \lambda ^2 t_a^2 \right) \nonumber \\&\quad = N \dfrac{\lambda ^2 t_a^2}{2}. \end{aligned}$$

To get an intuition of the magnitude of these numbers, consider, for instance, an input spike train lasting 1 s with presynaptic spike rate of $0.5\,$Hz which generates two postsynaptic spikes and we pick the relevant time window to be twice $\tau _S$, so $l=2$ and $s_{\mathrm{post}} = 3$. Then, the expected number of events of type (1) would be

37 $$\begin{aligned} {\mathbb {E}}\left[ \#1\right] \approx \dfrac{M}{400}. \end{aligned}$$

Furthermore, not all of those events would actually be problematic; if all of them are potentiating or depressing, then this does not change our analysis.

For case (2) we argue that in order to spontaneously generate new spikes, the synapses affected by STDP must be very strong and excitatory, and a few of those strong excitatory synapses must coincide within a small time window of order $\tau _m$.

The synapses that can be very strong are those in the $t_a$ time, meaning that we expect

38 $$\begin{aligned} n_a = \rho t_a = \rho l\tau _S s_{\mathrm{post}}, \end{aligned}$$

independent synapses to be close to $w_{\max }$. Each one of those synapses can fire within the remaining $T-t_a$ time at a rate $\lambda$, so we would expect to have a presynaptic rate of STDP-affected spikes of

39 $$\begin{aligned} \lambda _{a} = n_a \lambda . \end{aligned}$$

Now, we must compute the probability that enough of them coincide to generate a postsynaptic spike.

We denote this number by *k* and we will compute the number of spontaneous postsynaptic spikes that would appear for every *k*. We start by considering $k=2$ of those presynaptic spikes (although for some choices of $w_{\max }$ we have to start at $k>2$), and note that in order to have the postsynaptic spike, we must have

40 $$\begin{aligned} w_{\max } + w_{\max }\mathrm{e}^{-\frac{\Delta t_{k=2}}{\tau _m}} + \sigma _v > v_{th} \end{aligned}$$

where $\sigma _v$ is a term that accounts for the presence of other spikes that could be driving the membrane potential higher, and $\Delta t_{k=2}$ is the time interval between the two spikes. By rearranging,

41 $$\begin{aligned} \Delta t_{k=2} < i_{2} = \tau _m \ln \left( \vartheta - 1\right) , \end{aligned}$$

where $\vartheta = \frac{v_{th} - \sigma _v}{w_{\max }}$. Since the spikes follow a Poisson distribution, the probability of a time interval between spikes is given by an exponential distribution, so

42 $$\begin{aligned} \text {Pr}\left[ \#2|k=2\right] = \text {Pr}\left[ \Delta t_{k=2} < i_{2}\right] = 1 - \mathrm{e}^{-\lambda _a i_2}, \end{aligned}$$

and the number of those intervals tends to $\lambda _a T$ for large *T*, so

43 $$\begin{aligned} {\mathbb {E}}\left[ \#2\right] _{k=2} = \lambda _a T \left( 1 - \mathrm{e}^{-\lambda _a i_2}\right) , \end{aligned}$$

For $k>2$, the estimation can be done by applying the fact that two contiguous spikes are independent, and therefore, the inter-spike intervals are also independent, so we can multiply their probabilities. Furthermore, we should not have any two spikes at a distance closer than $i_2$, so

44 $$\begin{aligned} \text {Pr}\left[ \#2|k=3\right]< & {} \int _{i_2}^{\infty } \lambda _a \mathrm{e}^{-\lambda _ax} \int _{i_2}^{\infty } \lambda _a \mathrm{e}^{-\lambda _ay} \Theta \nonumber \\&\quad \left[ 1 + \mathrm{e}^{-\frac{x}{\tau _m}} + \mathrm{e}^{-\frac{y}{\tau _m}} - \vartheta \right] \mathrm{d}y \mathrm{d}x, \end{aligned}$$

where the inequality comes because we let the interval time go to infinity, while *T* is finite. We can ignore the $\Theta \left[ 1 + \mathrm{e}^{-\frac{x}{\tau _m}} + \mathrm{e}^{-\frac{y}{\tau _m}} - \vartheta \right]$ term and we obtain

45 $$\begin{aligned} \text {Pr}\left[ \#2|k=3\right] < \left( 1-\mathrm{e}^{-\lambda _a i_2}\right) ^2. \end{aligned}$$

And here the number of pairs of contiguous time intervals is also lower than $T\lambda _a$, which gives us

46 $$\begin{aligned} {\mathbb {E}}\left[ \#2\right] _{k=3} \le T\lambda _a \left( 1-\mathrm{e}^{-\lambda _a i_2}\right) ^2. \end{aligned}$$

Naturally, the same upper bound can be computed for any *k*, so

47 $$\begin{aligned} \begin{aligned} {\mathbb {E}}\left[ \#2\right] _{\forall k}&= \sum _{j=2}^{\infty } {\mathbb {E}}\left[ \#2\right] _{k=j}\le T\lambda _a \sum _{j=2}^{\infty }\left( 1-\mathrm{e}^{-\lambda _a i_2}\right) ^{j-1}\\&= T\lambda _a \mathrm{e}^{\lambda _a i_2} = T\lambda _a \left( \vartheta - 1\right) ^{\lambda _a}. \end{aligned} \end{aligned}$$

Which will be low as long as $\lambda _a$ is low. If we have, for instance, $M=50$, $l=2$, $s_{\mathrm{post}}=3$ and $\lambda = 0.5\,$Hz, we obtain $\lambda _a = 6 \cdot 10^{-3}$. Then, if we take $\sigma _v= w_{\max }/2$, $\vartheta = 1.5$,

48 $$\begin{aligned} \begin{aligned} {\mathbb {E}}\left[ \#2\right] _{\forall k} \approx \dfrac{3T}{1000}, \end{aligned} \end{aligned}$$

with *T* being in milliseconds, this means that for an input spike train lasting half a second, generating 3 postsynaptic spikes, there would be one expected spontaneous postsynaptic spike.

The estimates from Eqs. 37 and 48 give a relatively low number of coupled postsynaptic spikes or spontaneous spikes. We will therefore assume, from now on, that the effects described in Sect. 3 are valid and happen in every postsynaptic spike on every neuron independently of the presence of other postsynaptic spikes.

## Spike trains subject to alternative plasticity rules

Here, we simulate the effects of other learning rules on a spike train to compare it with the rule that we proposed. Although we will keep similar parameters for the initialization, input rate, fraction of neurons and probability of a random input, we will vary some of the STDP parameters.

Another family of variants comes from the review by (Vogels et al. 2013) mentioned earlier, where different inhibitory rules are discussed. We will not comment on the rules that require firing bursts or subthreshold induction protocols, as those are outside of the scope of this paper, and we will also not comment on the kernels that only dampen inhibition, because those require some other mechanism to prevent inhibition from being effectively depressed. Finally, we will also ignore the inhibition kernels that have long time constants $\tau _s^i\approx 100 ms$ because those are already on the scale of the stimulus length.

Explicitly we will address the following variants:

1. Altering the STDP time constant $\tau _s^e$ to be twice as large for the inhibitory weight $\tau _s^i$ than for the excitatory one. This makes inhibition grow faster than excitation, breaking the symmetry between the excitatory and inhibitory weight distributions and hence giving us a much lower firing rate, although the advance of the first spikes is still visible.
2. To make a fair comparison we take again the same rule with the parameters of the inhibition to be the same as for excitation, so that $A_+^i = A_+^e$ and $w_{\max }^e = w_{\max }^i$. This leads to the CDF of the first spike dominating being larger than the CDF of the subsequent spikes for most of the time interval, thus pushing the first spike forward, although the difference in instantaneous rates is difficult to see.
3. As many studies now have found that pairs of excitatory spikes are not enough to characterize the change in weights, we also study triplet rules. Given our low rate assumption—see “Appendix A”—we do not need to worry about pairs of presynaptic spikes, so we will take one of the most well-known versions of it that considers two postsynaptic spikes where the weight change in the second is modulated by the distance to the first (Gjorgjieva et al. 2011). We find that our results are mostly unchanged, as the first postsynaptic spike remains unchanged and the others would at most reduce their probability.
4. Another common variation of excitatory plasticity is to add a homeostatic term that penalizes large weights proportionally with the output firing rate of a neuron. This ensures that any neuron keeps a moderated rate, and thus reduces the strength of the excitatory part balancing the potentiation of the STDP. However, we found again that our results remain similar, as the global weight reduction does not change the fact that the first spike moves forward.
5. Regarding inhibitory plasticity, the first kernel that we try is to reverse the STDP, so that synapses whose presynaptic spikes arrive after the postsynaptic spike are potentiating and those that arrive before are depressed. As this rule goes into the same direction as excitatory STDP, it promotes the same effect that purely excitatory STDP or the balanced case would, only more.
6. Finally, the last variation that we will try is to use a mexican hat kernel for inhibitory plasticity. What we do find is that the results agree with our previous result.

We present the simulation results in Fig. 6. We can easily observe that most of the rules exposed here do support our conclusions, even if an analytical approach is not straightforward.

## Recurrent connections

Although in this work we have assumed that every neuron gets a fixed spike train, the existence of connections between neurons implies that when one neuron changes its postsynaptic spike train the presynaptic input of its neighbors might change. Hence, as opposed to the random but uniformly distributed input that we used, neurons might receive a non-uniform input. While we will not address this case in detail, we will assess by simulations whether our results should remain valid.

An important assumption when dealing with recurrent connections is that the network must remain stable. This is necessary in many contexts to prevent runaway behavior, and in our case will help us assess the final results.

The first result that differs from our previous discussion is that the inter-spike intervals are shorter than the delay to the first spike, even before the STDP appears. This comes from the fact that neurons can trigger each other’s activity, hence a subset of neurons will fire much more often than the equivalent population with no recurrent connections, hence shortening the inter-spike-intervals (Fig. 7).

The effect of the STDP on the first spike is similar to the one we described, as it pushes the first spike forward in time. However, for secondary spikes they also get pushed forward, probably because the recurrent connections get severely reinforced by triggering spikes: if a pair—or a cycle of any length—of neurons excite each other, they continue to be active and reinforce their spikes to their maximum. This also implies that the number of spikes grows, as the inter-spike intervals grow shorter.

Naturally, the effects that differ from our previous results such as changes in the number of spikes depend on the strength of the recurrent connections; there is a continuum of possible values, from fully feed-forward architectures to strongly recurrent ones as we have simulated here, and the increase or decrease in the number of spikes depends on which effect dominates—either the decrease in purely feed-forward or the increase in strongly recurrent.
